# Proteome Response of *Staphylococcus xylosus* DSM 20266T to Anaerobiosis and Nitrite Exposure

**DOI:** 10.3389/fmicb.2018.02275

**Published:** 2018-09-25

**Authors:** Laura Quintieri, Marzia Giribaldi, Maria Gabriella Giuffrida, Teresa Maria Creanza, Nicola Ancona, Laura Cavallarin, Maria De Angelis, Leonardo Caputo

**Affiliations:** ^1^Institute of Sciences of Food Production, National Research Council of Italy, Bari, Italy; ^2^Institute of Sciences of Food Production, National Research Council of Italy, Turin, Italy; ^3^Consiglio per la Ricerca in Agricoltura e l’Analisi dell’Economia Agraria, Centro di Ricerca in Ingegneria e Trasformazioni Agroalimentari, Turin, Italy; ^4^Istituto di Sistemi e Tecnologie Industriali Intelligenti per il Manifatturiero Avanzato (STIIMA), National Research Council, Bari, Italy; ^5^Department of Soil, Plant and Food Science, University of Bari Aldo Moro, Bari, Italy

**Keywords:** meat starter, curing agents, adaptive responses, 2DE/MALDI-TOF/TOF-MS, KEGG enrichment, phenotypic microarray

## Abstract

The viability and competitiveness of *Staphylococcus xylosus* in meat mostly depend on the ability to adapt itself to rapid oxygen and nutrients depletion during meat fermentation. The utilization of nitrite instead of oxygen becomes a successful strategy for this strain to improve its performance in anaerobiosis; however, metabolic pathways of this strain underlying this adaptation, are partially known. The aim of this study was to provide an overview on proteomic changes of *S. xylosus* DSM 20266T cultured under anaerobiosis and nitrite exposure. Thus, two different cultures of this strain, supplemented or not with nitrite, were *in vitro* incubated in aerobiosis and anaerobiosis monitoring cell viability, pH, oxidation reduction potential and nitrite content. Protein extracts, obtained from cells, collected as nitrite content was depleted, were analyzed by 2DE/MALDI-TOF/TOF-MS. Results showed that DSM 20266T growth was significantly sustained by nitrite in anaerobiosis, whereas no differences were found in aerobiosis. Accordingly, nitrite content was depleted after 13 h only in anaerobiosis. At this time of sampling, a comparative proteomic analysis showed 45 differentially expressed proteins. Most differences were found between aerobic and anaerobic cultures without nitrite; the induction of glycolytic enzymes and glyoxylate cycle, the reduction of TCA enzymes, and acetate fermentation were found in anaerobiosis to produce ATP and maintain the cell redox balance. In anaerobic cultures the nitrite supplementation partially restored TCA cycle, and reduced the amount of glycolytic enzymes. These results were confirmed by phenotypic microarray that, for the first time, was carried out on cell previously adapted at the different growth conditions. Overall, metabolic changes were similar between aerobiosis and anaerobiosis NO_2_-adapted cells, whilst cells grown under anaerobiosis showed different assimilation profiles by confirming proteomic data; indeed, these latter extensively assimilated substrates addressed at both supplying glucose for glycolysis or fueling alternative pathways to TCA cycle. In conclusion, metabolic pathways underlying the ability of *S. xylosus* to adapt itself to oxygen starvation were revealed; the addition of nitrite allowed *S. xylosus* to take advantage of nitrite to this condition, restoring some metabolic pathway underlying aerobic behavior of the strain.

## Introduction

*Staphylococcus xylosus* is one of the main coagulase-negative staphylococci species found in most traditionally cured meat products (Talon and Leroy, 2006; Fiorentini et al., 2009) and naturally occurred on human and animal skin (Azelmad et al., 2017). Due to their well-studied characteristics, *S. xylosus* strains have long been used as starter culture in the production of fermented sausages together with other microorganisms (Di Maria et al., 2002; Di Cagno et al., 2008; Caputo et al., 2011). Indeed, they contribute to the flavor development of meat products *via* their catabolism of branched-chain amino acids in odorous volatile methyl compounds; they avoid rancidity by their antioxidant properties and they favor buttery aroma by their catabolism of pyruvate in diacetyl and acetoin (Barrière et al., 2001; Olesen and Stahnke, 2004). *S. xylosus* has been also shown to convert metmyoglobin to nitrosomyoglobin in culture medium, in salami and in raw meat, without addition of nitrate or nitrite (Morita et al., 1998; Li et al., 2013; Ras et al., 2017). However, *S. xylosu*s and *Staphylococcus carnosus* were also able to reduce the residual amounts of nitrates and nitrites (Neubauer and Götz, 1996; Gøtterup et al., 2007; Mah and Hwang, 2009; Bosse et al., 2016).

Nitrate and/or nitrite salts constitute the curing agents in the formulation of most fermented sausages and many processed meat products. They are chiefly used to enhance the color and flavor and inhibit the growth of numerous bacteria such as *Clostridia* (Roncalés, 2015). However, high residual concentrations of these curing agents in dry fermented sausages may cause the development of the carcinogenic nitrosamines during gastric digestion (Oostindjer et al., 2014). Thus, their levels in meat products were limited by European Commission (EU Commission, 2011) and by other International Agencies.

The reduction of nitrite amount is preferentially promoted by oxygen depletion as nitrate and nitrite act as alternative electron acceptors supporting the anaerobic respiration and growth, as reported in *S. carnosus* (Neubauer and Götz, 1996). Indeed, under oxygen depletion the ability to use nitrogen compounds showed a significant increase in growth of *S. carnosus* and *S. xylosus* in comparison with the same culture grown in defined medium without nitrite (Neubauer and Götz, 1996; Bosse et al., 2016). Likewise, the load of *S. xylosus*, inoculated in salami Milano, was higher in presence of nitrite in comparison with the meat sample without curing agents; catalase activity and reduction of nitrite and nitrate amounts also contributed to sensory and physicochemical properties of products (Fiorentini et al., 2010).

During fermentation of meat products microbial starters, such as *S. xylosus*, should rely on several strategies in order to tackle dramatic reduction in oxygen and nutrient availability (Brown, 2000) and counteract competitiveness of other naturally occurred microorganisms. Indeed, food system and specifically the conditions used in salami manufacturing process (i.e., drying, salting and addition of nitrate/nitrite salts, fermentation causing acidification) can be considered a stressful environment that microorganisms, such as *S. xylosus*, overcome by developing a network of metabolic activities also positively affecting organoleptic properties and health attributes of final product (Guerzoni, 2010). Therefore, the understanding of these dynamic stress responses is crucial to select stable starter and to obtain desirable and safe products (Guerzoni, 2010; Suzzi, 2011).

In the last decades, proteomics has substantially contributed to understand cellular mechanisms of individual organisms offering excellent possibilities to probe many protein functions and response under different *stimuli*, such as oxidative stresses caused by the presence of reactive oxygen and nitrogen species (ROS and RNS). Even though the technological properties of *S. xylosus* strains are widely characterized in meat, the metabolic pathways for its adaptation under oxygen depletion and nitrite/nitrate amount need to be further investigated (Mainar et al., 2014; Vermassen et al., 2014).

Moreover, proteomic data can be integrated with phenotype microarray in order to better understand or validate predicted metabolic pathways involved in the physiological response to different metabolites and environments (Kirubakar et al., 2018).

Recently, a strain of *S. xylosus* was inoculated at high concentrations in a meat batter containing both nitrate and nitrite and a transcriptomic approach was performed under microaerophilic conditions (Vermassen et al., 2014). Results highlighted that curing agents were principally involved in the modulation of the expression of genes responsible for overcoming nitrosative stress in meat. However, these authors did not assess the oxygen concentration during fermentation of meat batter. Moreover, this latter growth parameter was found to affect biochemical features of *S. xylosus* more than curing agents and sugar (Stahnke, 1999).

Other authors also demonstrated that in a closest species (*S. carnosus*) nitrate is converted to nitrite to generate a proton motive force that allows these bacteria to grow anaerobically. When the nitrate pool is depleted, the growth of such bacteria under these conditions and in the presence of nitrite is justified with the reactivation of metabolic mechanisms based on the increase of NAD^+^/NADH ratio (Neubauer and Götz, 1996; Schlag et al., 2008). However, to the best of our knowledge, the metabolic response of *S. xylosus* to nitrite alone in relation to oxygen depletion needs to be further elucidated.

Therefore, in this study we aim to shed light on the main pathways involved in adaptability of *S. xylosus* DSM 20266T, in oxygen starvation and in presence of nitrite. Even though this strain was previously isolated by human skin (Schleifer and Kloos, 1975), it was added during fermentation and ripening of soppressata Molisana exhibiting a behavior as a meat starter (Di Maria et al., 2002).

The comparative proteomic analysis was performed on this strain cultured under aerobiosis and anaerobiosis and nitrite supplementation to induce low (aerobic) and high (anaerobic) nitrite consumption. Proteomic data, also evaluated through KEGG (Kyoto Encyclopedia of Genes and Genomes) enrichment analysis, revealed that the alternative utilization of nitrite under anaerobiosis restored some metabolic pathways underlying aerobic behavior of the strain; these data were validated by Biolog phenotype microarray tests carried out on cells previously grown at each incubation condition.

## Materials and Methods

### Media and Culture Conditions

*Staphylococcus xylosus* DSM 20266T (type strain), purchased from Leibniz Institute DSMZ-German Collection of Microorganisms and Cell Cultures (DSMZ, Braunschweig, Germany) was grown overnight in Nutrient Broth (Oxoid S.p.A., Milan, Italy) at 37°C under shaking conditions (170 strokes per min), and diluted in sterile saline solution to reach 0.153 ± 0.05 of optical density (OD) at 600 nm (corresponding to *ca.* 7 log cfu mL^-1^).

Then, an appropriate volume of this diluted bacterial suspension was inoculated (*ca.* 3 log cfu mL^-1^) in a final volume of 100 mL of Basic Medium broth (BM; Neubauer and Götz, 1996), supplemented or not with 150 ppm of NaNO_2_. Three biological replicates from each culture were performed. All samples were incubated in both aerobiosis and anaerobiosis. In particular, the cultures were aerobically grown in 1,000 mL Erlenmeyer flasks covered with cotton plugs whilst the anaerobic conditions were performed in 100 mL screw-cap bottles after adding to culture medium 0.5 g L^-1^ of sodium thioglycolate (Sigma-Aldrich s.r.l., Milan, Italy) as reducing agent (Evans and Kloos, 1972). Aerobic and anaerobic cultures were incubated on a rotary shaker at 160 strokes/min and 37°C. Bacterial growth was monitored evaluating the OD changes up to 24 h in comparison to those of un-inoculated BM broth.

### Microbiological Counts and Analytical Determinations

At different interval times (3, 7, 11, 12, 13, 16, and 24 h), viable cell counts (log cfu mL^-1^, for each biological replicate) were evaluated by plating decimally diluted cultures in sterile saline solution (0.9% NaCl), on Petri dishes of BM agar (BM supplemented with 1.6% of agar), in triplicate. Nitrite concentration was colorimetrically determined on supernatants of each culture, recovered by centrifugation at 8,000 × *g* for 10 min at 4°C, following the method of Griess (Association of Official Analytical Chemists [AOAC], 2005). In addition, the values of pH and oxidation reduction potential (ORP) were monitored with the pH 50 benchtop pH meter equipped with both pH and ORP electrodes (XS Instruments, Carpi, Modena, Italy). Three replicates were considered for each determination. OD values of microbial cultures were tested for homogeneity by Levene’s test before carrying out ANOVA by using IMB–SPSS statistic software version 20 (IMB corp., Chicago, IL, United States). Then, a three-way ANOVA was performed by the Univariate General Linear Model (GLM) procedure to analyze the independent effects of incubation times, nitrite content in culture medium and incubation conditions (aerobiosis/anaerobiosis) on OD changes of *S. xylosus* DSM 20266T. Partial eta-squared analysis was performed to assess the effect sizes of each main factor and the related interactions. Multiple comparison test among all individual means was made by Fisher’s least significant difference (LSD) test at the 95% confidence interval. Differences between viable cell counts (log cfu mL^-1^) and physico-chemical parameters (pH and ORP) at 0 and 13 h of incubation were evaluated by Tukey HSD multiple comparison test (*P* < 0.05).

### Proteomic Analysis

Proteomic analyses were performed on cells recovered from each culture at 13 h of incubation; this time of sampling was chosen on the basis of the complete consumption of nitrite concentration.

Thus, after centrifugation at 7,000 × *g* at for 10 min, cell pellets were washed with PBS (Sigma-Aldrich s.r.l.) twice. Cell lysis was carried out with 40 μL of lysostaphin (0.622 UμL^-1^; lysostaphin from *Staphylococcus staphylolyticus* ≥ 500 units mg^-1^ protein; L7386 Sigma-Aldrich s.r.l.) in accordance with the Schindler and Schuhardt (1964) procedure with minor modifications. Briefly, each pellet was re-suspended in PBS to reach an OD value of 2.4 ± 0.24. Then, 40 μL of lysostaphin from *S. staphylolyticus* and 4 μL of Benzonase (0.0379 U/μL) were added to each sample, and incubated for 15 min at 37°C. After lysis, each sample was centrifuged (10 min at 16,000 × *g* at 4°C) and 100 μL of protease inhibitor cocktail (Sigma-Aldrich s.r.l.) was added to each supernatant. Protein concentration of each cell protein extract was determined in triplicate with Bradford reagent (Bio-Rad Laboratories, Milan, Italy).

Each protein extract (50 μg) was purified with the 2-DE Clean up kit, following manufacturer’s instructions. Each sample (from three biological replicates × four treatments), was then re-suspended with 200 μL of IPG rehydration buffer (7M urea, 2M thiourea, 66 mM DTT, 2% CHAPS, 0.5% ampholytes), and loaded, in triplicate, on immobilized pH gradient strips (11.0 cm × 3.3 × 0.5 mm, linear pH range: 4–7). IPG strips were actively rehydrated for 12 h at 50 V and 20°C. Isoelectrofocusing was carried out on PROTEAN IEF Cell until 53,500 Vh, starting with a voltage of 500 V for 1 h, then 1,000 V for 1 h and finally up to 8,000 V for 6.5 h. IEF strips were equilibrated in reduction buffer (50 mM Tris–HCl pH 8.8, 6 M urea, 30% glycerol, 2% SDS, 0.002% bromophenol blue and 10 mM DTT) for 15 min and then in alkylation buffer (50 mM Tris–HCl pH 8.8, 6 M urea, 30% glycerol, 2% SDS, 0.002% bromophenol blue and 135 mM iodoacetamide) for 15 min.

The equilibrated gel strips were embedded at the top of SDS-PAGE gels (16 × 18 × 0.1 cm, T 12%, C 3%) in molten 1% agarose as described previously (Planchon et al., 2009). Gels, (three gels for each biological replicate) were stained with freshly prepared Blue Colloidal Coomassie stain (Candiano et al., 2004) and scanned with a GS-800 calibrated Densitometer (Bio-Rad). The best gel images (from a total of 36 excluding 1 gel × treatment), showing total gel density nearest to the average value, were included in image analysis by Image Master^TM^ 2D Platinum 7.0 software (GE Healthcare). The gel with the highest spot-count and the best resolved spots, as detected by spot detection parameters (3-smooth, 8-saliency, 5-min area), was set as the master gel. All gel images were automatically matched to the master gel, then subsequent manual editing was carried out to correct both false-positive and negative matches. To ensure normalization of spot quantities, protein spot densities were normalized (% V) on total volumes of all the spots in each gel image (De Angelis et al., 2008).

Principal component analysis (PCA) was performed by using IBM-SPSS software v 20.0 (IBM, Armonk, NY, United States) in order to cluster normalized spot volume data differentially expressed in *S. xylosus* DSM 20266T in relation to nitrite supplementation and incubation conditions. In addition, the multiple range Tukey’s test and independent-sample *t*-test at the 95% confidence interval were used to detect significant differences in volume percent among protein spots of the same matches but from different gel classes. Protein spots with a fold change ≥ 2, *P* < 0.05 and a minimum 0.1% V were arbitrarily selected and excised from the gel for their identification. Spots were cut out from the 2DE gels, destained overnight (with a solution of 50 mM ammonium bicarbonate and 40% ethanol), washed three times for 10 min with acetonitrile and then dried in a Speedvac. Proteins were in gel digested with trypsin (Promega, Madison, WI, United States), and spectra were acquired as previously described (Zava et al., 2009). The selected spots were identified by means of MALDI-TOF/TOF mass spectrometry, using an Ultraflex II MALDI-TOF/TOF instrument (Bruker Daltonics, Germany). Evaluation of the mass spectra and the generation of the peak list were obtained using Flex Analysis software (Bruker Daltonik, Germany). Peaks were de-isotoped and background peaks were identified and removed by the software. The tandem mass spectra were acquired on the same mass spectrometer, run LID experiments using LIFT TOF/TOF acquisition. The MS/MS spectra were automatically analyzed by Flex Analysis software (Bruker Daltonik, Germany). The MS-Fit and MS-Tag software packages v 5.18.1^1^ were used to search against the NCBI 2018.1.18 database by using the peptide mass fingerprinting method (PMF; Pappin et al., 1993). The following search parameters were set for the searches: Taxonomy search on *S. xylosus* (12771 entries), *S*-carbamidomethyl derivate on cysteine as fixed modification, oxidation on methionine as variable modification and three missed cleavage sites for trypsin digestion. The peptide mass tolerance was 20 ppm. The identified proteins were classified on the basis of their biological functions, by using the bioinformatic resource Uniprot.

Metabolic pathways mainly affected by the aerobiosis condition and the nitrite addition were also evaluated by KEGG enrichment pathway analysis (Bove et al., 2012). The enrichment *p*-values were evaluated by using the Fisher’s exact test. These statistical analyses were carried out by using MATLAB statistics toolbox.

### Biolog Phenotype Microarrays

In order to complement proteomic analysis, Biolog phenotype array test (Biolog, Inc., Hayward, CA, United States) was also carried out to compare phenotypic profiles of *S. xylosus* DSM 20266T cells; in particular cells were cultivated, in triplicate, under the experimental conditions causing significant proteome changes.

Tests were performed using the Biolog 96-well AN MicroPlate^TM^ according to the manufacturer’s instructions. Briefly, wells of Biolog plates were inoculated with 150 μL of each bacterial suspensions, obtained under the selected experimental condition and adjusted to 65% transmittance as recommended by the manufacturer. The plates were incubated at 37°C for 72 h. Endpoint reads were automatically recorded by using a Microplate Reader (Biolog) with 590 nm and 750 nm wavelength filters at 24-h time intervals. Positive records were obtained by subtracting absorbance readings at 590 nm from those registered at 750 nm which corrects for any background light scattering.

## Results and Discussion

### Effect of Anaerobiosis and Nitrite Exposure on the Bacterial Growth

**FIGURE 1:**
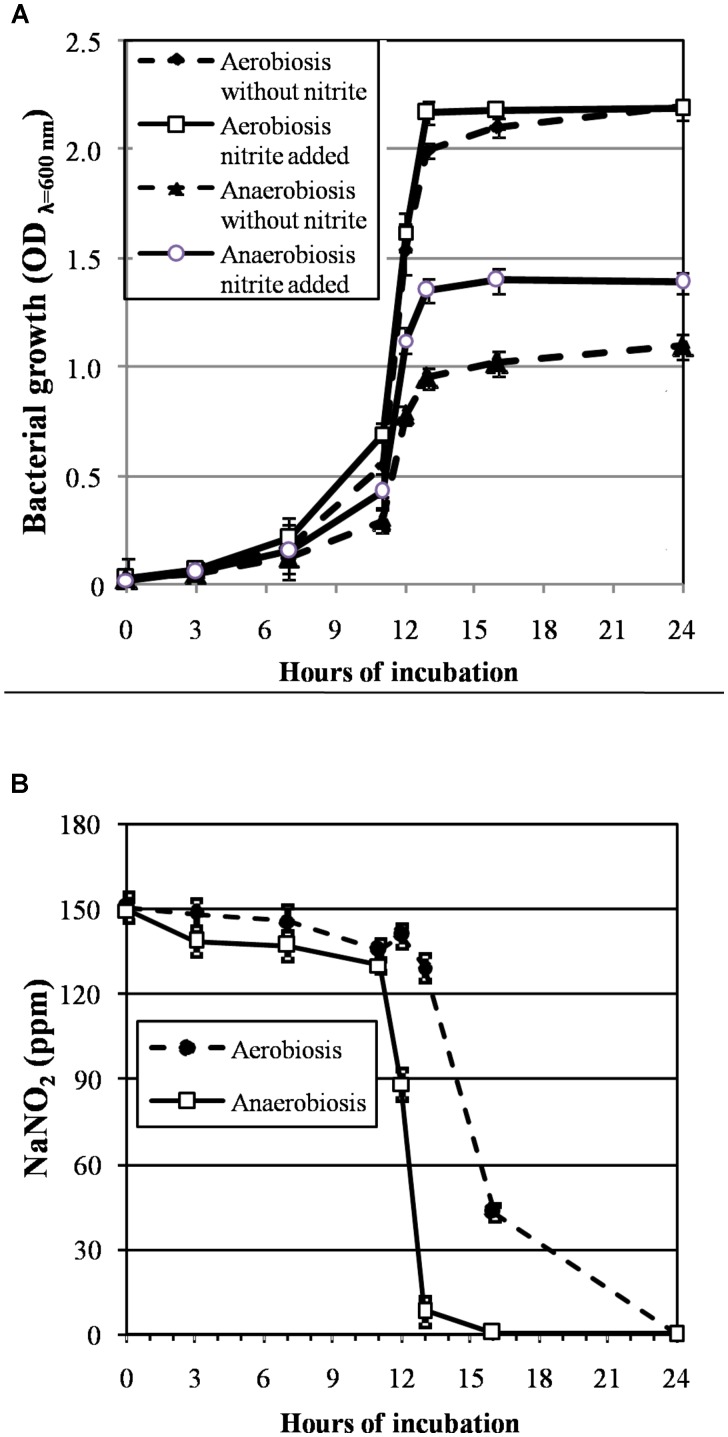
**(A)** Growth curves (OD_600nm_) of *S. xylosus* DSM 20266T under aerobic and anaerobic conditions for 24 h in basic medium (BM) containing or not sodium nitrite (150 ppm). Each value represents the average ± standard deviation (bars). Differences among all values higher than 0.244 OD_600nm_ are considered statistically significant according to *post hoc* multi-comparison Fisher’s least significant difference test (95% interval confidence). **(B)** Reduction of nitrite content (ppm NaNO_2_) in supernatant of *S. xylosus* DSM 20266T cultures during growth under different conditions. Each value represents the average ± standard deviation (bars).

The growth curves of *S. xylosus* DSM 20266T, expressed as time-dependent OD increases, showed a lag phase within first 3 h of incubation, registering OD value that ranged from 0.013 to 0.031, on average (corresponding to cell density from *ca*. 3 log to 3.5 log cfu mL^-1^, respectively); then, the OD increased to reach an average OD value of 0.10 at 7 h of incubation; this latter value was similar in each culture (*P* > 0.05) and corresponded to *ca*. 4.8 log cfu mL^-1^ (**Figure 1A**). Afterward, the OD of aerobic and anaerobic cultures was affected by interaction between nitrite supplementation and incubation time (**Figure 1A**). In particular, both the three-way (incubation time × growth condition × nitrite amendment) and the two-way interaction terms tested (incubation time × growth condition, time × nitrite amendment and growth condition × nitrite amendment) were found to be highly significant (*P* < 0.01). The main effects contributing to the variability of OD_600nm_ of the strain under the experimental conditions were associated to the incubation time, growth condition, incubation time × growth condition and nitrite amendment as shown by the Partial eta-squared analysis. Simple main effect analyses showed that after 13 h of incubation *S. xylosus* DSM 20266T density increased more rapidly under aerobic conditions than under anaerobic conditions reaching values by average of 2.080 and 1.154 OD_600nm_, respectively (corresponding to *ca*. 9.3 and 6.5 log cfu mL^-1^, on average, respectively; **Figure 1A** and **Table 1**). After this time no significant changes in OD values and cell density were registered for each culture (**Figure 1A**).

**Table 1:** Viable cell counts (log cfu mL^-1^) and physico-chemical parameters (pH and oxidation-reduction potential, ORP) of *S. xylosus* DSM 20266T cultures at the beginning and at the end (13 h) of incubation in basic medium under different conditions.

Cultures	Time (hours)	Log cfu mL^-1^	pH	ORP (mV)
Aerobiosis without nitrite	0	3.35 ± 0.20^d^	7.80 ± 0.15^a^	178.0 ± 4.5^a^
	13	9.36 ± 0.35^a^	7.12 ± 0.02^b^	21.7 ± 10.5^b^
Aerobiosis with nitrite	0	3.25 ± 0.30^d^	7.78 ± 0.17^a^	151.0 ± 11.3^a^
	13	9.28 ± 0.34^a^	7.19 ± 0.03^b^	29.7 ± 17.1^b^
Anaerobiosis without nitrite	0	3.0 ± 0.15^d^	7.64 ± 0.27^a^	-135.0 ± 6.5^c^
	13	6.06 ± 0.05^c^	6.93 ± 0.02^b^	-122.3 ± 11.3^c^
Anaerobiosis with nitrite	0	3.28 ± 0.14^d^	7.73 ± 0.14^a^	-132.0 ± 9.9^c^
	13	6.98 ± 0.17^b^	6.95 ± 0.02^b^	-110 ± 11.6^c^


*Each value represents the average ± standard deviation (*n* = 3). Means of each sample with same superscript letters in column do not differ significantly (*P* > 0.05; Tukey HSD multiple comparison test).*

By summarizing, under aerobiosis no significant (*P* > 0.05) differences in OD values were found in relation to nitrite supplementation throughout all the incubation period; conversely, under anaerobiosis cultures lately boosted their growth in presence of nitrite (**Figure 1A**).

Likewise, the initial nitrite concentration of anaerobic cultures registered a significant reduction at 12 h of incubation (**Figure 1B**). In contrast, no significant change in nitrite concentration was found in aerobic cultures throughout 12 h of incubation. However, after 13 h of incubation under aerobiosis with or without nitrite, the redox potential of culture medium significantly (*P* < 0.05) decreased by an average of 139 mV (**Table 1**); in concomitant with this ORP decrease the nitrite content significantly (*P* < 0.05) dropped by an average of 14.20% (**Figure 1B**).

A weak acidification was instead observed in all samples at 13-h incubation (**Table 1**).

These results were consistent with those found by Neubauer and Götz (1996) in anaerobic cultures of *S. carnosus* that showed in presence of nitrite a growth rate significantly higher than that found in medium without nitrite; indeed, under anaerobiosis, nitrate and nitrite, were used as terminal electron acceptors and coupled to the generation of a proton motive force, which is directly utilized as a source of energy or transformed into ATP by a membrane-associated ATPase (Unden and Bongaerts, 1997). However, in our experimental conditions, nitrite depletion rate in these latter cultures was higher than that found by the same authors.

Therefore, in anaerobiosis DSM 20266T seemed to take advantage of nitrite probably involved in a modification of metabolic pathways. In order to identify more specific protein targets in the response mechanisms of *S. xylosus* DSM 20266T to these experimental conditions, cells from each 13-h culture were recovered and analyzed by using a proteomic approach.

### Comparative Proteomic Analysis

In our work, we compared proteome patterns shown by 13-h cultures of *S. xylosus* DSM 20266T in response to oxygen and nitrite changes in order to assign the affected pathways upon these stimuli.

**FIGURE 2:**
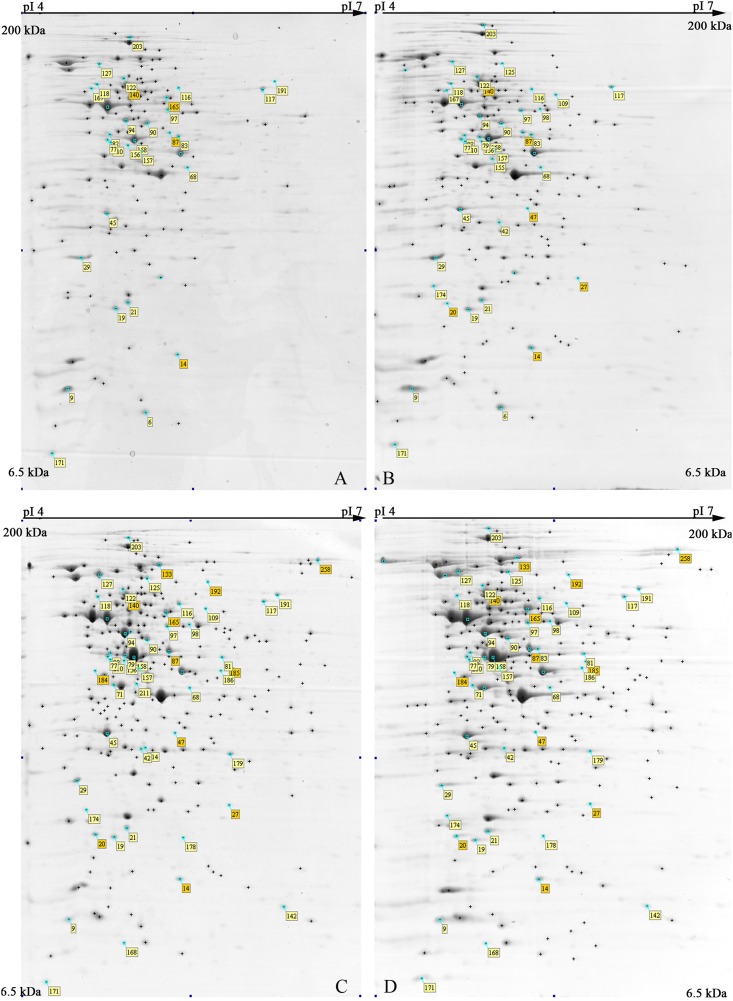
Representative 2-DE maps of *S. xylosus* DSM 20266T grown in different conditions for 13 h: **(A)** synthetic medium in aerobiosis; **(B)** synthetic medium added with nitrite in aerobiosis; **(C)** synthetic medium in anaerobiosis; **(D)** synthetic medium added with nitrite in anaerobiosis. Protein spots showing significantly different intensities (*P* < 0.05;≥ of twofold; min Vol. 0.1%) are labeled by the corresponding match number in the square box, while yellow and orange marked identified and not identified proteins, respectively.

The 2-DE maps of *S. xylosus* proteome, extracted from each culture, resulted in 250 ± 15 protein spots (**Figure 2**); scatter plots of spot values from each pair of gels showed a correlation coefficient of 0.98, on average.

Principal component analysis was applied to examine global differences in proteins from *S. xylosus* DSM 20266T cultures with respect to oxygen and nitrite presence (**Supplementary Figure S1**). The analysis revealed that the almost all variance of protein dataset was explained by the first 19 components (PCs); however, only the first three PCs explaining *ca.* 50% of the total variance were taken in account since they were able to effectively separate the original dataset into three main groups (**Supplementary Figure S1**). Indeed, 31.77% of the variance was explained by the first principal component (PC1) grouping bacterial cultures in relation to oxygen depletion; the second and third components (PC2 and PC3), explaining 21.31% of variance, allowed to segregate only anaerobic cultures with respect of nitrite supplementation; by contrast, spot matches of aerobic cultures were not affected by nitrite presence.

These results agreed with those reported for *S. aureus* (Fuchs et al., 2010). In their study, PCA analysis showed a similarity between protein synthesis patterns of cells grown under anaerobic conditions and those grown anaerobically in the presence of nitrate as electron acceptor. The role of oxygen in the modulation of *S. xylosus* metabolism were suggested by Stahnke (1999), even before. Indeed, this author reported that oxygen in general had more influence on the aroma producing capacity of *S. xylosus* than of *S. carnosus* which was more affected by nitrate and glucose.

In order to shed light on *S. xylosus* protein changes and metabolic pathways affected by both by oxygen and nitrite a total of 55 spots, displaying significant changes (*P* < 0.05) of more than twofold in relative abundance, were selected as differentially expressed spots; among these 45 proteins were identified by MS and discussed below.

### Effect of Anaerobiosis on *Staphylococcus xylosus* Proteome

**FIGURE 3:**
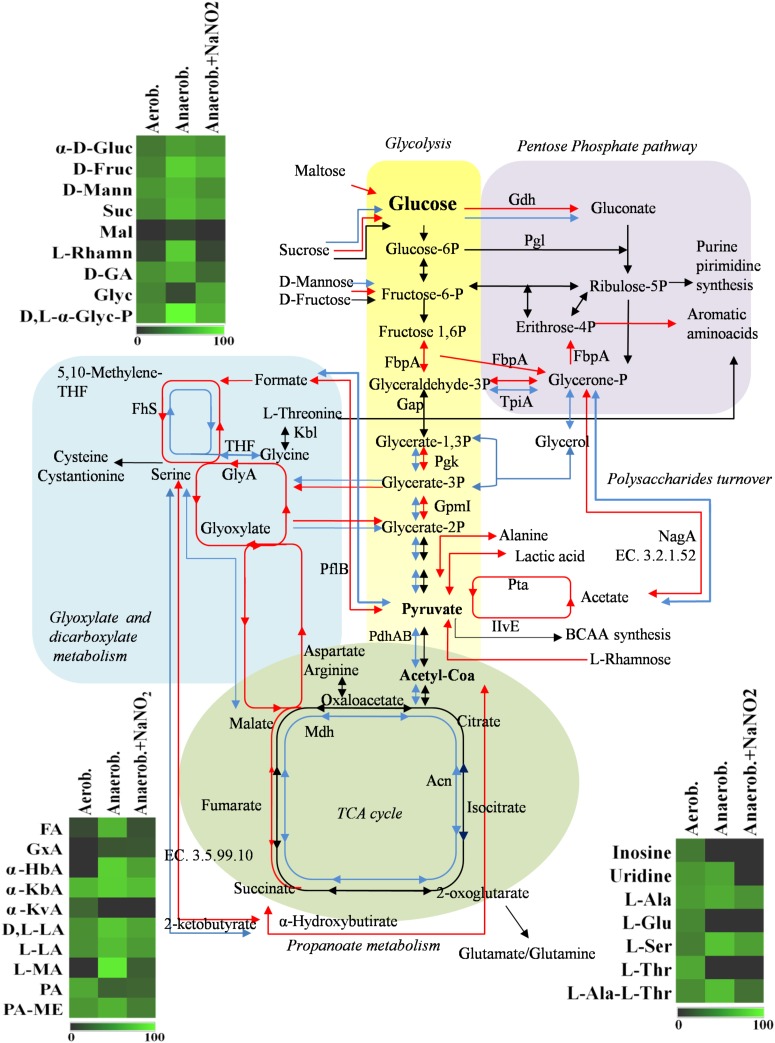
Schematic representation of changes in DSM 20266T metabolism. Colored arrows and enzymes indicate metabolic pathways induced (false discovery rate, FDR < 0.05) under anaerobiosis without nitrite (red line) or under anaerobiosis in presence of nitrite (blue line) in comparison to metabolic pathways detected under aerobiosis (dark line). Heatmap inserts compare phenotypic microarrays based on assimilation substrate by DSM 20266T strain upon different growth conditions. The legend of color code from black to dark green, and chartreuse green indicate low, moderate, and high utilization of carbon and nitrogen sources, respectively, assessed as arbitrary Biolog values. Substrates: FA, fumaric acid; GA: glyoxylic acid; α-HbA, α-hydroxybutyric acid; α-KbA, α-ketobutyric acid; α-KvA, α-ketovaleric acid; D,L-LA, D,L-lactic acid; L-LA, L-lactic acid; MA, L-malic acid; PA, pyruvic acid; PA-ME, pyruvic acid methyl ester; α-D-Gluc, α-D-glucose; D-Fruc, D-fructose; D-Mann, D-mannose; Mal, maltose; D-GA, D-gluconic acid; Glyc, glycerol; D,L-α-Glyc-P, D,L-α-glycerol-P; Inosine, inosine; Uridine, uridine; L-Ala, L-alanine; L-Glu, L-glutamic acid; L-Ser, L-serine; L-Thr, L-threonine; L-Ala-L-Thr, L-alanyl-L-threonine; Enzymes: Gdh, sugar dehydrogenase; TpiA, triosephosphate isomerize; Gap, glyceraldehyde-3-phosphate dehydrogenase; IlvE, branched-chain-amino-acid aminotransferase; MetB, cystathionine gamma-synthase; PhdA, pyruvate dehydrogenase E1 component alpha subunit; PdhB, pyruvate dehydrogenase E1 component beta subunit; Pgk, phosphoglycerate kinase; Kbl, glycine *C*-acetyltransferase; GlyA, serine hydroxymethyltransferase; GpmI, phosphoglyceromutase; Mdh, malate dehydrogenase; Pgl, 6-phosphogluconolactonase; Fhs, formate-tetrahydrofolate ligase; NagA, *N*-acetylglucosamine-6-phosphate deacetylase; Acn, aconitate hydratase; Pta, phosphate acetyltransferase; FbpA, fructose-bisphosphate aldolase; PflB, formate *C*-acetyltransferase; EC 3.5.99.10 RidA family protein; EC 3.2.1.52 Beta-hexosaminidase.

The adaptation to each incubation condition was characterized by changes in some metabolic pathways as depicted in **Figure 3**. Under oxygen depletion protein changes were correlated to energy metabolism (glycolysis and TCA cycle; **Table 2**), cell wall turnover, protein and nucleotide synthesis. Indeed, TpiA, GpmI, Pgk, FbpA were induced, indicating enhanced glycolytic activity in anaerobiosis. By contrast, the pyruvate dehydrogenase complex (PdhA1) and TCA enzymes were repressed or reduced under anaerobic condition (**Table 2** and **Supplementary Table S1**).

The phosphate acetyltransferase protein (Pta), involved in the conversion of acetyl-CoA acetate and ATP, was detected only under anaerobic condition (**Table 2** and **Supplementary Table S1**). In addition to this, we exclusively detected the formate C-acetyl transferase (PflB) under anaerobiosis. As demonstrated for other microorganisms, PlfB is a ubiquitous oxygen-sensitive enzyme supporting the production of an extra ATP molecule through acetate assimilation and/or formate fixation (Zelcbuch et al., 2016). The over expression of formate/nitrite transporters and *pfl* genes was also found in a meat model inoculated with *S. xylosus* and simulating osmotic stress establishing under the fermentation step (Vermassen et al., 2016). Therefore, in accordance with this study, our data suggested the establishment of a fermentative pyruvate-related pathway involved in production of acetate by glycolytic activity. Likewise, Ferreira et al. (2013) reported that, during the anaerobic growth, *S. aureus* COL-S strain used glucose at a rate higher than in presence of oxygen; lactate was the main end-product, but minor amounts of acetate, ethanol and 2,3-butanediol were also formed. Moreover, a shotgun metagenomics of the microbiota of sausages fermented with *Lactobacillus sakei* and *S. xylosus* revealed genes associated with the reduction of acetaldehyde to ethanol and acetyl phosphate to acetate and 2,3-butanediol to acetoin (Ferrocino et al., 2018). In *S. xylosus* DSM 20266T the absence of oxygen prevents carbon flow through the TCA cycle, decreasing the concentrations of biosynthetic intermediates (such α-ketoglutarate, oxaloacetate) and consequently amino acids biosynthesis, such as glutamate/glutamine and aspartate/asparagine (**Figure 3**). By contrast, the synthesis of aromatic amino acids was promoted from pentose phosphate intermediate (erythrose-4P; Richardson et al., 2015). Anaerobiosis also determined the increase in the synthesis of enzymes correlated to one carbon metabolism and serine/glycine biosynthesis (FhS, GlyA); these pathways were putatively sustained by the increased glycolytic intermediate (glycerate-3P) as well as by the formate, in turn obtained from pyruvate (**Figure 3**; Wendrich and Marahiel, 1997). The one-carbon metabolism includes the reactions whereby one-carbon units are transferred, *via* tetrahydrofolate-derivatives, from the donors serine, glycine, or formate to essential biosynthetic processes (nucleotides, vitamins, and some amino acids); serine and glycine are reversibly converted each other *via* the cytoplasmic serine hydroxymethyl transferase (GlyA). Enzymatic reactions involved in folate-intermediates make a major contribution to NADPH production and formate detoxification (Leibig et al., 2011; Sah et al., 2015); in other microorganisms (*Saccharomyces cerevisiae* and *E. coli*) the one-carbon metabolism was also implicated in the cellular response to a shift to anaerobiosis; the varied cellular pathways included the hierarchical serine utilization direct to cell wall protein biosynthesis rather than over other proteins (Tsoi et al., 2009).

In anaerobic DSM 20266T cells the occurrence of the enzyme 4-hydroxy-tetrahydrodipicolinate reductase (DapB) suggested a possible involvement of pyruvate in L-lysine biosynthesis, in turn correlated with protein and cell-wall peptidoglycan synthesis (Dogovski et al., 2012). These latter pathways were also sustained in anaerobiosis by the increase in volume percent of the enzyme glucosamine-fructose-6-phosphate aminotransferase (GlmS) correlated with the amino sugar biosynthesis (**Table 2**). In addition, the membrane protein *N*-acetylglucosamine-6-phosphate deacetylase (NagA) involved in the carbohydrate metabolism was induced to yield glucosamine-6-phosphate and acetate under the same conditions (**Table 2** and **Supplementary Table S1**). Together with the NagA increase we also found the induction of GpsB (**Table 2** and **Supplementary Table S1**) involved in the formation of peptidoglycan cross-links. Then, these results suggested the hypothesis of a turnover of the newly synthesized peptidoglycan as already described for *S. aureus* and *Bacillus subtilis* (Claessen et al., 2008; Reith and Mayer, 2011); this hypothesis was also reinforced by the anaerobiosis up-regulated beta-hexosaminidase, an acetyl hexosaminidase, in turn correlated to biofilm detachment (Zhu et al., 2018).

**Table 2:** Fold changes of *Staphylococcus xylosus* DSM 20266T proteins synthesized under the different experimental conditions (ANitrite/A, aerobiosis with nitrite/aerobiosis; ANA/A, anaerobiosis/aerobiosis; ANANitrite/A, anaerobiosis with nitrite/aerobiosis; ANAnitrite/ANA, anaerobiosis with nitrite/anaerobiosis; ≥2-fold, *P* < 0.05).

Match ID	Accession^a^	Functional classification^b^	Protein annotation	Symbol	Fold changes			
					
					ANitrite/A	ANA/A	ANANitrite/A	ANANitrite/ANA
45	WP_029377651	Glycolysis/Gluconeogenesis	Triosephosphate isomerase	TpiA	1.18	2.04	1.66	0.81
77	AID01643.1	Glycolysis	Glyceraldehyde-3-phosphate dehydrogenase	Gap	1.05	0.50	0.50	0.96
94	WP_029377652	Glycolysis	Phosphoglycerate kinase	Pgk	1.25	2.64	3.22	1.22
127	WP_029377650	Glycolysis	Phosphoglyceromutase	GpmI	0.92	2.10	1.51	0.72
211	WP_029377997	Glycolysis/gluconeogenesis	Fructose-bisphosphate aldolase	FpbA	n.d.^c^	ON^1^	n.d.	OFF^2^
184	WP_017724146	Glycolysis	Pyruvate dehydrogenase E1 component beta subunit	PdhB	n.d.	ON^1^	ON^2^	2.70
90	CEF19225	Glycolysis	Pyruvate dehydrogenase E1 component alpha subunit	PhdA1	0.95	0.25	0.41	1.63
155	WP_029377731	Tricarboxylic acid (TCA) cycle	Malate dehydrogenase	Mdh	1.16	OFF^1^	0.56	ON^2^
203	WP_042362767	Tricarboxylic acid (TCA) cycle	Aconitate hydratase	Acn	1.21	0.28	0.23	0.83
157	AID42338	Pentose phosphate pathway	6-Phosphogluconolactonase	Pgl	0.97	0.43	OFF.	OFF^2^
42	WP_029378512	Pentose phosphate pathway	Sugar dehydrogenase	Gdh	n.d.	ON^1^	ON^2^	1.30
125	WP_042362290	Carbohydrate metabolism	Glucosamine-fructose-6-phosphate aminotransferase	GlmS	1.21	2.43	2.93	1.21
71	WP_042362747	Proteolysis	M42 peptidase		1.38	4.31	2.62	0.61
109	WP_042363020	Proteolysis	Leucyl aminopeptidase family protein	PepB	0.75	OFF^1^	0.50	ON^2^
158	WP_042362628	Proteolysis	Aminopeptidase P family protein	PepP	1.08	2.11	1.54	0.73
122	WP_042363396	Glutamine metabolic process	GMP synthase	GuaA	1.00	0.35	0.45	1.29
79	WP_029379088	AMP biosynthesis; purine nucleobase biosynthetic process	Phosphoribosylamine-glycine ligase	PurD	1.54	5.11	4.43	0.87
81	WP_017723470	AMP biosynthesis via *de novo* pathway; purine nucleobase biosynthetic process	Adenylosuccinate lyase	PurB	1.09	2.09	2.55	1.22
98	WP_029377986	Glyoxylate and dicarboxylate metabolism	Serine hydroxymethyl transferase	GlyA	1.21	2.07	2.29	1.10
82	AID43584	Amino acid metabolism	BCAA aminotransferase	IlvE	0.76	0.48	0.39	0.82
314	CEF18861	Amino acid metabolism	4-Hydroxy-tetrahydrodipicolinate reductase	DapB	n.d.	ON^1^	n.d.	OFF^2^
83	CEF17712	Amino acid metabolism	Cystathionine gamma-synthase	MetB	0.95	OFF^1^	OFF^1^	n.d.
97	WP_029377923	Amino acid metabolism	Glycine *C*-acetyltransferase	Kbl	1.26	0.50	0.93	1.79
6	WP_017723387	Translation	30S ribosomal protein S6	RpsF	1.09	0.34	0.50	1.46
21	WP_042363303	Translation	50S ribosomal protein L10	RplJ	0.97	0.42	0.63	1.52
167	WP_029377464	Transcription	Transcription termination protein NusA	NusA	0.94	0.31	0.47	1.50
27	WP_041079558	Transcription	Transcription termination/anti-termination protein NusG	NusG	0.84	0.33	0.52	1.56
9	AID01344	Transcription	Cold shock protein CspA		1.13	0.23	0.42	1.82
174	WP_029377137	Transcription	Transcription elongation factor GreA	GreA	0.72	0.42	0.60	1.41
156	WP_029378572	Response to oxidative stress	Rhodanese domain-containing protein		1.04	OFF^1^	0.46	ON^2^
29	WP_019469891	Response to oxidative stress	Peroxiredoxin	AhpC	1.17	0.38	0.32	0.83
118	WP_039069254	Response to oxidative stress	Alkyl hydroperoxide reductase subunit F	AhpF	0.92	0.41	0.28	0.69
171	AID02929	Stress response	Cold shock protein		1.35	0.28	0.91	3.23
178	WP_029377419	Response to oxidative stress	Glutathione peroxidase		1.39	ON^1^	ON^2^	1.42
179	WP_017724290	Response to oxidative stress	GTP-sensing pleiotropic transcriptional regulator CodY		n.d.	ON^1^	ON^2^	0.59
192	WP_019470080	Polysaccharides turnover	Beta-hexosaminidase		0.73	2.18	1.06	0.49
332	WP_042363127	Carbohydrate metabolism/polysaccharides turnover	*N*-Acetylglucosamine-6-phosphate deacetylase	NagA	n.d.	ON^1^	ON^2^	0.29
168	WP_029379204	Metabolic process	RidA family protein		n.d.	ON^1^	ON^2^	1.18
191	WP_042363540	Glyoxylate and dicarboxylate metabolism	Formate-tetrahydrofolate ligase	FhS	n.d.	ON^1^	ON^2^	1.24
210	A7KJJ6	Pyruvate metabolism/fermentative pathways	Phosphate acetyl transferase	Pta	n.d.	ON^1^	n.d.	OFF^2^
186	WP_042363535	Metabolic process	Acyl-CoA dehydrogenase		n.d.	n.d.	ON^2^	ON^2^
258	WP_038678780	Glucose metabolic process	Formate *C*-acetyl transferase	PflB	n.d.	ON^1^	ON^2^	0.63
68	WP_017723057	Cofactor biosynthesis	Pyridoxal 5^′^-phosphate synthase lyase subunit PdxS		0.88	OFF^1^	0.58	n.d.
116	WP_038677953	Protein biosynthesis	Cysteinyl-tRNA synthetase	CysS	0.88	2.69	0.94	0.35
142	WP_029377274	Cell cycle, cell division	Cell division protein GpsB	GpsB	OFF^1^	2.55	1.45	0.57


*^*a*^NCBI classification; ^*b*^Uniprot database; ^*c*^n.d., not detected in the analyzed conditions; ^*1*^ON exclusively identified under anaerobiosis; ^*2*^ON exclusively identified under anaerobiosis with nitrite; ^*1*^OFF exclusively identified under aerobiosis; ^*2*^OFF exclusively identified under anaerobiosis.*

Under anaerobic conditions the enzymes, involved in *de novo* purine/pyrimidine (Pgl) and protein biosynthesis such as ribosomal proteins RpsF, ribosomal proteins 50S, and ribosomal proteins L10, and the transcription anti-termination proteins NusA and NusG were down regulated. These results were also in accordance with previous studies (Wendrich and Marahiel, 1997; Fuchs et al., 2007). In contrast to the inhibition of protein biosynthesis, in the anaerobic cells the amount of proteolytic enzyme, peptidase M42 and PepP, significantly increased to putatively sustain the intracellular pool of amino acids (glutamate, aspartate, glutamine, and proline). These enzymes could be involved in the proteolytic activity of staphylococci addressed to the release of free amino acids and peptides in fermented meat products, as previously reported (Roncalés, 2015). In accordance with this hypothesis glutamate and glutamine were previously found in high concentration in the meat model inoculated with *S. xylosus* by Vermassen et al. (2016).

As here reported, *S. xylosus* DSM 20266T was able to rearrange metabolic pathways in order to survive and grow also in the absence of alternative terminal acceptors (O_2_ or NO_2-_). Under anaerobiosis, the pleiotropic transcriptional regulator CodY was detected. This result partially agreed with those found in *S. aureus* (Zühlke et al., 2016); indeed in this strain, although CodY was detected also under aerobiosis, its synthesis registered a strong level increase in response to oxygen starvation. The metabolic pathways regulated by CodY, directly or indirectly, include those for amino acid biosynthetic pathways (typically BCAA, threonine, arginine, glutamate, and histidine), purine biosynthesis (particularly the steps from IMP to GMP), the Krebs cycle, sugar and amino acid transport, carbon overflow metabolism, chemotaxis and motility. Except for carbon overflow, most of these pathways are repressed by CodY (Richardson et al., 2015), in accordance with results described above.

The adaptation of bacterial cells to stresses (osmotic, oxidative, acid, etc.) is associated with several protective mechanisms, also referred to as “cross-protection”(Capozzi et al., 2009); in order to overcome the deleterious effects of oxidative and nitrosative stress, staphylococci have evolved protection, detoxification, and repair mechanisms (Gaupp et al., 2012; Caballero et al., 2018). In our work, detoxifying enzymes (peroxiredoxin, AhpC/AhpF system, and the rhodanese domain-containing protein) and the cold shock protein CspA registered higher amount under aerobiosis than under anaerobiosis; in particular, these proteins are grouped among the cellular defense mechanisms against endogenous oxidative stress induced by the aerobic respiration; occasionally, the incomplete reduction of oxygen to H_2_O can generate ROS (endogenous superoxide anions, O_2-_, and hydrogen peroxide, H_2_O_2_) by the interaction with flavoproteins (e.g., oxidases and monooxygenases). Therefore, DSM 20266T synthesizes peroxiredoxins and the disulfide reductase AhpC/AhpF system to detoxify alkyl hydroperoxides by converting them to their corresponding alcohols using NADH or NADPH as the reducing equivalents (Gaupp et al., 2012). By contrast, the synthesis of the glutathione peroxide was exclusively detected under anaerobic condition in accordance with previous studies (Resch et al., 2005).

Main pathways affected by oxygen depletion were also revealed by an enrichment analysis of KEGG pathways performed between aerobic and anaerobic conditions (**Figure 3**). As already discussed, these results confirmed that in anaerobic cells, glucose was metabolized through an increased activity of glycolytic enzymes and, as Krebs cycle was reduced, the accumulated pyruvate was converted to acetate through an alternative pathway of carbon fixation. To this regard the up-regulated glycolytic enzyme TpiA and the down-regulated Mdh, PhdAB were common to most affected pathways, underlying their important role in the change of *S. xylosus* metabolism under oxygen-limiting conditions.

### Effect of Nitrite Supplementation on *S. xylosus* Proteome

Under aerobic condition the nitrite addition did not significantly affected *S. xylosus* proteome (**Table 2** and **Supplementary Table S1**); these results agreed with the similar growth pattern of the strain. In contrast, the amounts of enzymes shared by anaerobic cultures with or without nitrite were significantly different (*P* < 0.009). Metabolic pathways positively affected by nitrite supplementation were shown in **Figure 3**.

Under anaerobiosis with nitrite supplementation glycolytic enzyme amounts dropped, whilst those correlated to TCA cycle increased (**Figure 3**). These results agreed with a previous study reporting the importance of pyruvate dehydrogenase (Pdh) up-regulation for maintenance of normal TCA cycle turnover in *Achromobacter denitrificans* in the presence of nitrite (Doi et al., 2014).

The occurrence of acyl-CoA dehydrogenase was exclusively found in cells grown under anaerobic condition combined with nitrite addition (**Table 2**); this enzyme probably favored β-oxidation of fatty acids as fueling source and the biosynthesis of membranes (Richardson et al., 2015). Moreover, this protein was responsible to the alkylation response protein AidB. This latter, induced by alkylating agents, anaerobiosis, and acetate at acidic pH, neutralized nitrosoguanidines or their intermediates that could damage cells (Landini et al., 1994).

It has been previously demonstrated that in anaerobiosis the nitrite reduction by *Staphylococcus* spp. was hypothesized to be correlated with the activation of the membrane bound nitrate reductase of the NarG type (*nar*GHJI genes) and the cytoplasmic nitrite reductase (*nir*RBD genes), respectively (Schlag et al., 2008). However, transcripts of the *nar* and *nir* operons, coding for nitrate and nitrite reductases, respectively, were found to be expressed at elevated levels under anaerobic conditions also in the absence of alternative electron acceptors, such as nitrate (Fedtke et al., 2002; Vermassen et al., 2016). This was of particular interest since nitrate seems to be unnecessary for the anaerobic expression of these genes. Unfortunately, proteomic analysis did not revealed these enzymes, even though in both anaerobic cultures supplemented or not with nitrite, we detected CodY regulating the synthesis of nitrite-transport proteins (Pohl et al., 2009).

The nitrite metabolism is correlated with the activation/repression of O_2_ dependent or redox sensors, such as NreABC and Rex- NAD^+^/NADH sensors, respectively. In particular, Rex senses changes of the NADH/NAD^+^ ratio in the cytosol and is active as repressor in the presence of NAD^+^ and inactivated by NADH. Under aerobic conditions, the NAD^+^/NADH ratio rises due to rapid oxidation of NADH by the electron flow through the respiratory chain. By contrast, under anaerobic conditions, NADH levels increase and Rex repressor activity is inactivated (McLaughlin et al., 2010). The Rex repressor inactivation up-regulated pathways involved in fermentation (lactate, formate, and ethanol), nitrate/nitrite respiration and represses the SsrAB promoter of TCA cycle (Pagels et al., 2010); this was in accordance with our results. Recently, Chaudhari et al. (2017) demonstrated that by a unknown mechanism low nitrite concentrations can activate respiration by affecting cytochrome b-oxidase and restoring NAD^+^. This could explain protein changes (such as Pta, Pfl) detected under anaerobiosis and nitrite supplementation in comparison to anaerobiosis. KEGG enrichment analysis confirmed that in presence of nitrite *S. xylosus* DSM 20266T rearranged some of metabolic KEGG pathways, previously affected under anaerobiosis (**Figure 3**). Among these latter, DSM 20266T partially re-established the energetic cycle as the TCA cycle and the reduction of fermentative pathways catalyzed by Pta enzyme. By contrast, carbon metabolism was sustained by increased level of Pga and GmpI and putatively fuelled by glycerone 3-P, in turn synthesized from polysaccharides degradation. The addition of nitrite did not affected glyoxylate and dicarboxylate that was rather favored by oxygen depletion.

### Phenotypic Microarray Analysis

**FIGURE 4:**
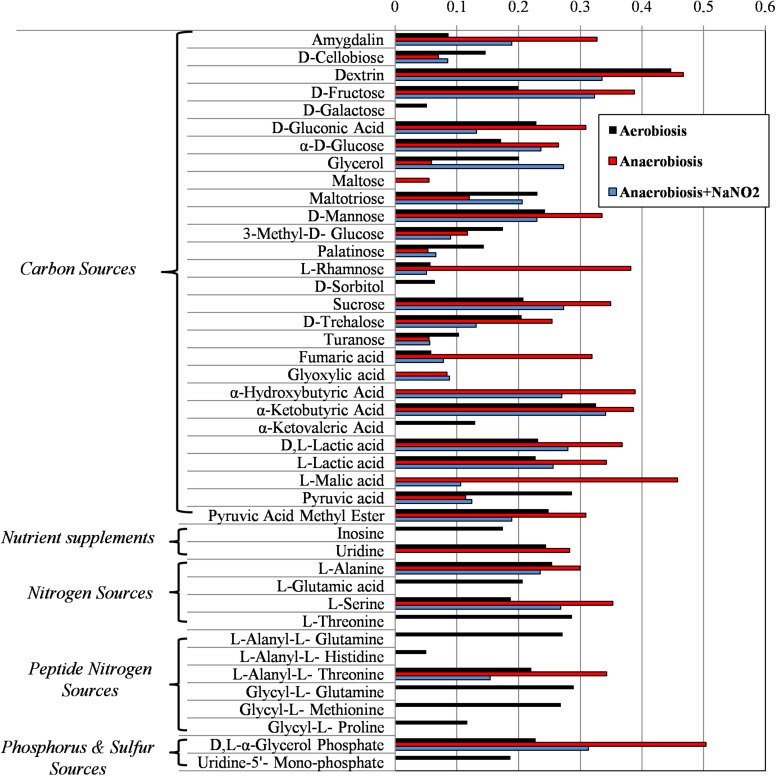
Biolog phenotype microarray of *S. xylosus* DSM 20266T cells from 13 h cultures under aerobiosis or anaerobiosis or anaerobiosis in synthetic medium added with nitrite.

Results from microbiological and proteomic analyses highlighted that main protein changes were found among three experimental conditions: aerobiosis without nitrite supplementation and anaerobiosis with or without nitrite. Therefore, *S. xylosus* DSM 20266T cells from 13-h cultures cultivated in these three conditions were evaluated for their metabolic fingerprints on AN Biolog plates (**Figure 4**). Results showed that cells exhibited different metabolic pattern; in particular aerobic-conditioned cells utilized a total of 38 out of 95 substrates. Conversely, 30 and 28 substrates were used by cells previously grown in anaerobiosis with or without nitrite, respectively. The substrates used by the DSM 20266T are shown in **Figures 3, 4**.

The usage of carbohydrates was more evident for bacterial cell previously grown under anaerobic conditions. Indeed, anaerobic cultures grown in the presence of 14 out 28 carbon sources showed absorbance values higher than 0.3.

In *S. xylosus* sugar catabolism is conducted by glycolysis and pentose phosphate pathway (Leroy et al., 2017). Therefore, under anaerobiosis in the DSM 20266T, sugars were metabolized by the enzymes involved in glycolysis (TpiA, Pgk, and GpmI), pentose phosphate pathway (PP; Gdh) registering increased levels in comparison with those found in aerobic conditions. Vermassen et al. (2016) suggested that *S. xylosus* catabolized glucose *via* the over-expressed Gdh to produce gluconate that in the PP were further metabolized to generate two NADH contributing to the redox status under anaerobic condition.

By contrast, the catabolism of most amino acids as secondary carbon sources decreased in anaerobic conditions.

Galactose was used only in cells from aerobic cultivation; this substrate is used to synthesize microbial polysaccharides, also bearing amino sugars, such as *N*-acetyl-glucosamine, *N*-acetyl-galactosamine. Interestingly, proteomic data from anaerobiosis cells suggested a cell wall turnover that, on this basis, could be unbalanced toward oligosaccharide hydrolysis with a presumptive reduction of the galactose uptake. Oddly, sorbitol, poorly assimilated by *S. xylosus* that instead prefers mannitol, was used only by aerobic cell of the DSM 20266T strain. Recent studies have also reported DNA sequences hexitol-specific PTS system (putatively associated with sorbitol dehydrogenase) in two *S. xylosus* strains isolated from mastitis and dermatitis (Dordet-Frisoni et al., 2007).

Among carbon source glycerol, poorly assimilated by *S. xylosus* upon anaerobiosis, was largely used by cells grown in anaerobiosis in presence of nitrite. Glycerol may be assimilated and phosphorylated to glyceraldehyde-3-phosphate (G3P). Then, G3P dehydrogenase (Gap) increased in cells grown in anaerobiosis and nitrite supplementation, usually catalyses the transfer of hydrogen from G3P generating NADH to NAD^+^ and then phosphorylates G3P to 1,3-bis-phosphoglycerate. The high glycerol assimilation in adapted cell grown in presence of nitrite agreed with other study; indeed, this latter reported a glycerol-dependent dose denitrification activity (Bernat et al., 2015).

Glyoxylate, α-hydroxybutyrate, and malate were mainly used by DSM 20266T cells grown under anaerobiosis with or without nitrite; however, by comparing these latter samples the assimilation of these compounds increased in cells grown under anaerobiosis without nitrite. Fumarate and α-ketobutyric acids were metabolized by all samples even though the related absorbance values were highest in cells grown under anaerobiosis without nitrite. These data integrated those from proteomic analyses suggest the partial re-activation of Krebs cycle (glyoxylate cycle) sustained by 2-ketobutyrate and α-hydroxybutyrate as by-products of propanoate metabolism; in addition, 2-ketobutyrate could be putatively obtained via the imino-deaminase WP_029379204 (EC. 3.5.99.10) from the amino acid serine. In order to devote carbon atoms for gluconeogenesis glyoxylate cycle utilizes acetate and fatty acids as carbon sources generating GTP and FADH. Due to the reactivation of TCA cycle under nitrite supplementation, this alternative pathway was consequently reduced.

An increase catabolism of alanine and L-alanyl-L-threonine was found in cells previously grown in anaerobic condition. Likewise, the concentration of alanine decreased under anaerobiosis in the meat model performed by Vermassen et al. (2016). Recently, this amino acid was found to be involved in the acetate production (Halsey et al., 2017). Indeed, glucogenic amino acids generating pyruvate (serine, threonine, glycine, and alanine) are important for ATP synthesis via substrate-level phosphorylation in the Pta/AckA pathway as also confirmed by proteomic data.

## Conclusion

During salami manufacturing, *S. xylosus* have to develop several physiologically adaptive responses to counteract the harsh conditions of process (acidification, oxygen starvation, oxidative stress) and then, to survive and growth. Among these, the transition from aerobic to the anaerobic conditions and the reduction of nitrate to nitrite, as found during sausage curing, is a serious challenge for the competitiveness and adaptability of *S. xylosus* DSM 20266T with a behavior similar to a meat starter. In our work *S. xylosus* strongly reduced its growth under anaerobiosis compared to that under aerobic conditions in synthetic medium; however, its adaptability to anaerobic condition was favored by its ability to rearrange some metabolic pathways in order to counteract the oxygen depletion. In particular, a total of 45 changed proteins were related to several important pathways. These results were confirmed and integrated with those obtained by phenotypic Biolog microarray performed on cells adapted at each grown condition. Thus, in DSM 20266T the lack of oxygen sustained activation of glycolysis, the block of TCA cycle, reduction of protein synthesis, the activation of glyoxylate cycle and acetate catabolism for the production of FADH/NADH and ATP. Catabolism of amino acids preferred gluconeogenic amino acids; by contrast most amino acids biosynthetic pathways were repressed. The nitrite supplementation markedly improved the growth rate of anaerobic cultures that in turn quickly consumed almost all nitrite content. Indeed, this latter by restoring some pathways, such as TCA cycle, reduced differences between cells grown under anaerobiosis and aerobiosis. Thus, the results of this work besides confirm DSM 20266T ability to cope the adverse effects of technological stress, occurring in meat environment, could be further exploited to increase safety and sensorial quality of sausages.

## Author Contributions

LQ and LeC designed the research and performed microbiological analysis. LQ, MG, and MGG performed proteomic analysis. LeC, NA, and TC performed statistical analysis and KEGG enrichment analysis. LeC and MA performed phenotype microarray. LQ wrote the paper. LeC, LaC, MGG, and MA revised the manuscript.

## Conflict of Interest Statement

The authors declare that the research was conducted in the absence of any commercial or financial relationships that could be construed as a potential conflict of interest.

## Footnotes

1 http://prospector.ucsf.edu

## Supplementary Material

FIGURE S1 Score plot obtained from principal component analysis (PCA) of two-dimensional electrophoresis (2DE) spot match dataset. Plot displays grouping of *S. xylosus* DSM 20226T cultures with respect of aerobiosis and anaerobiosis (open circle and square, respectively) along component 1 (PC1) and in relation to presence and absence of nitrite (filled circle and square, respectively) along components 2 and 3 (PC2 and PC3).

TABLE S1 Additional information for the differentially expressed proteins (*P* < 0.05; Fold > 2) listed in **Table 2**.

## Abbreviations

2DE: two-dimensional gel electrophoresis
MALDI-TOF/TOF-MS: matrix-assisted laser desorption ionization – time of flight mass tandem mass spectrometry
TCA cycle: tricarboxylic acid cycle
