# Supplementary figures and images for: Proteome Response of Staphylococcus xylosus DSM 20266T to Anaerobiosis and Nitrite Exposure
Source: Front Microbiol. 2018 Sep 25;9:2275. doi: 10.3389/fmicb.2018.02275 (PMC6167427; doi:10.3389/fmicb.2018.02275)

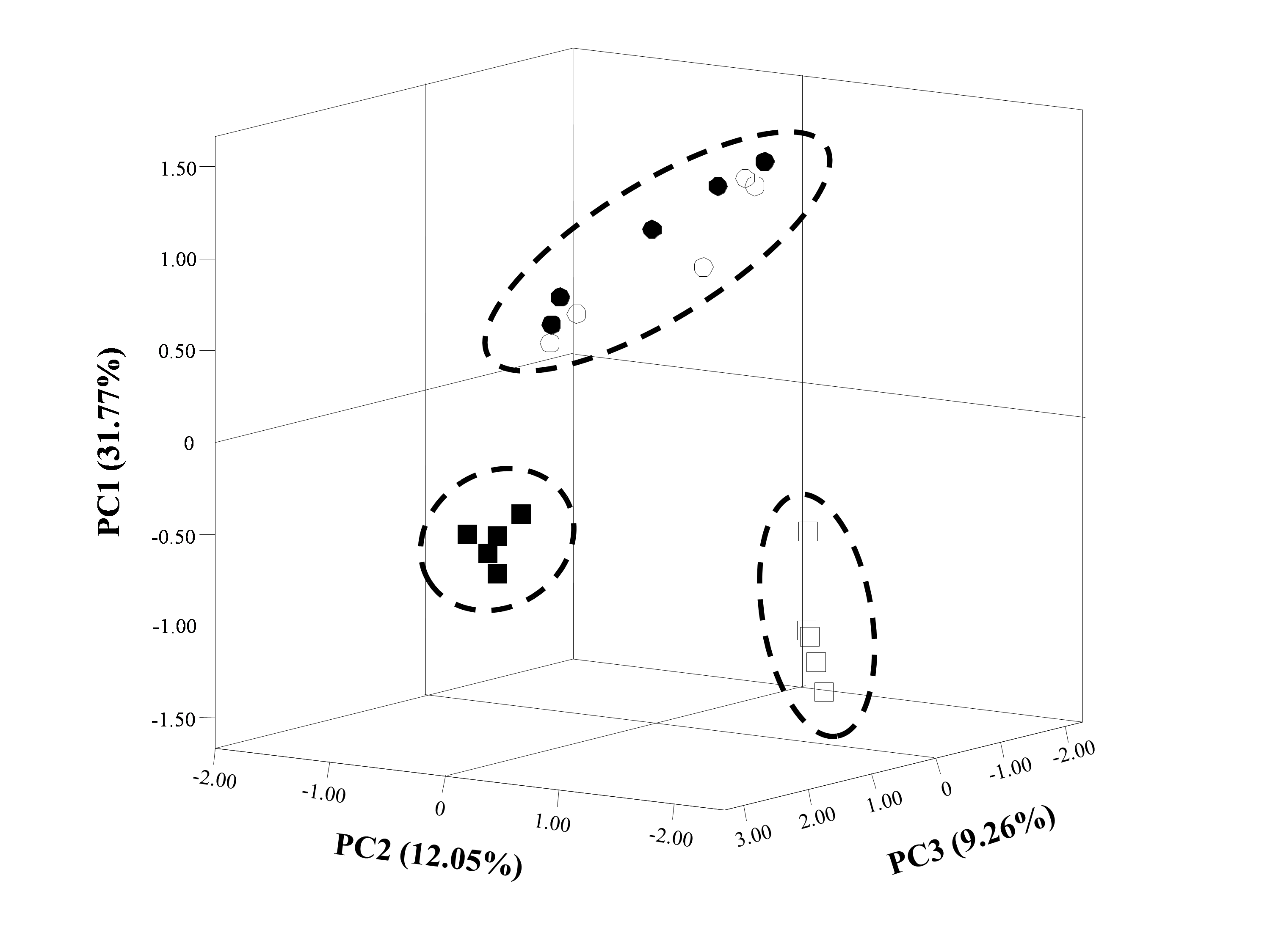

Supplement: FIGURE S1 — Score plot obtained from principal component analysis (PCA) of two-dimensional electrophoresis (2DE) spot match dataset. Plot displays grouping of S. xylosus DSM 20226T cultures with respect of aerobiosis and anaerobiosis (open circle and square, respectively) along component 1 (PC1) and in relation to presence and absence of nitrite (filled circle and square, respectively) along components 2 and 3 (PC2 and PC3). [file Image_1.PNG]
